# Type 2 Diabetes, PNPLA3 rs738409 Polymorphism, and the Risk of Liver Cirrhosis: Analysis of Taiwan Biobank

**DOI:** 10.3389/fgene.2022.822700

**Published:** 2022-03-07

**Authors:** Kuan-Chun Hsueh, Oswald Ndi Nfor, Shu-Yi Hsu, Shun-Fa Yang, Yung-Po Liaw

**Affiliations:** ^1^ Department of Surgery, Tungs’ Taichung Metro Harbor Hospital, Taichung, Taiwan; ^2^ Department of Post-Baccalaureate Medicine, College of Medicine, National Chung Hsing University, Taichung, Taiwan; ^3^ Department of Public Health and Institute of Public Health, Chung Shan Medical University, Taichung, Taiwan; ^4^ Institute of Medicine, Chung Shan Medical University, Taichung, Taiwan; ^5^ Department of Medical Research, Chung Shan Medical University Hospital, Taichung, Taiwan; ^6^ Department of Medical Imaging, Chung Shan Medical University Hospital, Taichung City, Taiwan

**Keywords:** liver, observational study, type 2 diabetes, polymorphism, chronic disease BUN (mg/dl) 0.996 0.954–1.040

## Abstract

Type 2 diabetes (T2D) and liver cirrhosis remain significant public health threats in Taiwan. These conditions are reported to be associated with the rs738409 polymorphism of the *patatin-like phospholipase domain-containing protein three gene (PNPLA3)* in European populations. We assessed the effect of T2D and *PNPLA3* rs738409 polymorphism on liver cirrhosis among Taiwan Biobank (TWB) participants. In total, 17,985 participants in TWB had their health records linked to the National Health Insurance Research Database (NHIRD). Participants included those who visited the assessment centers between 2008 and 2015, with an age range between 30 and 70 years of age. We performed logistic regression analysis to investigate the odds ratios (OR) for liver cirrhosis among participants based on the T2D status and rs738409 genotypes. Genotyping was performed using the Axiom Genome-Wide TWB Array Plate. In our analysis, 150 of the 17,619 eligible participants were identified as cirrhosis cases. Based on the univariate analysis, liver cirrhosis was positively associated with T2D (OR, 1.83; 95% CI 1.23–2.70) whereas, the variant rs738409 was not (regardless of the genetic model). The variant and T2D, however, showed significant interactions in the additive, genotype, and dominant models (*p* values of 0.0302, 0.0395, and 0.0455, respectively). We observed a statistically significant association between T2D and liver cirrhosis and variant rs738409 with an OR of 1.71 (95% CI, 1.03–2.84) for individuals carrying a G allele compared to those with a C allele and 2.92 (95% CI 1.07–7.99) for GG compared to CC individuals. According to our study, Taiwanese adults with T2D and the rs738409 GG genotype are more likely to develop liver cirrhosis.

## Introduction

Globally, liver diseases are one of the most important public health issues (Asrani et al., 2019). They remain a leading cause of morbidity and mortality. Cirrhosis is a potentially fatal complication of chronic liver disease, which may manifest 20–40 years after infection (Smith et al., 2019). It is the 11th leading cause of death globally (Asrani et al., 2019) and has a considerable economic impact (Smith et al., 2019). It is commonly caused by hepatitis, alcoholism, and non-alcoholic liver disease, but other causes remain unknown in approximately 5%–30% of those with this condition. (Nalbantoglu and Jain, 2019; Patel et al., 2019). As a result, exploring other possible variables may increase our understanding of the disease pathology.

Cirrhosis is strongly linked to viral hepatitis in several countries, including Taiwan (Mokdad et al., 2014; Sepanlou et al., 2020; Yang et al., 2020). In spite of this, the underlying causes and disease burden vary across demographic groups and geographic regions (Sepanlou et al., 2020). Diabetes is among the chronic conditions that have previously been linked to liver cirrhosis and mortality in various ethnic groups (Holstein et al., 2002; Quintana et al., 2016; Goh et al., 2017), even though the underlying pathogenic mechanisms are not entirely clear (Tolman et al., 2007). Mortality risk was found to be higher in patients with compensated liver cirrhosis who had T2D (Quintana et al., 2016).

Aside from the common causes, genetic factors have also been associated with liver cirrhosis (Stickel et al., 2020). However, the association between liver cirrhosis and a number of polymorphic variants has been inconsistent (Basyte-Bacevice et al., 2019; Chen et al., 2020). Among the previously identified polymorphisms, rs738409 variant of the *PNPLA3* gene has been strongly associated with cirrhosis and indices of liver damage, especially in Caucasians (Romeo et al., 2010). This variant has also been linked to liver-related events and death among Italians with nonalcoholic fatty liver disease (NAFLD) (Grimaudo et al., 2020), which is becoming more common in the eastern regions of Asia (Zhang et al., 2015). Despite its role in aggravating liver steatosis, the rs738409 variant has also been associated with a decreased risk of T2D in metabolically unhealthy middle-aged and elderly Chinese populations (Xia et al., 2019).

On the contrary, an earlier study found no association between diabetes and the PNPLA3 variant (Speliotes et al., 2010). Of note, diabetes and cirrhosis remain public health concerns in Taiwan. These two conditions have been associated with rs738409 polymorphism mainly in other populations, as cited above. Considering that the rs738409 variant has not been studied extensively among Asians, we examined the effect of this variant in combination with T2D on liver cirrhosis in TWB participants.

## Materials and Methods

### Data Sources

Through the data management system of the Health and Welfare Data Science Center (HWDC), we linked participants’ genotype data in TWB to their medical records in the NHIRD using personal identification numbers. Participants included those who visited the assessment centers between 2008 and 2015, with an age range between 30 and 70 years of age. Written informed consent was obtained from each participant before they were assessed. Disease information in the NHIRD was available from 1998 to 2015. Approval was obtained from the Institutional Review Board of Chung Shan Medical University. All authors had access to the study data and had reviewed and approved the final manuscript.

### Definition of the Variables

Data were available for 17,957 subjects in TWB. We excluded those with an incomplete questionnaire (*n* = 271) and genotyping information (*n* = 27), as well as cases of cirrhosis identified before the diagnosis of diabetes (*n* = 40). Finally, we identified 150 participants as cirrhosis cases and 17,469 as control individuals. Liver cirrhosis was diagnosed based on the International Classification of Diseases, Ninth Revision, Clinical Modification (ICD-9-CM), codes 571.2, 571.5, 571.6, 572.2, 572.3, 572.4, 572.8, 573.0) and either one-time hospitalization or two outpatient visits. Detailed information on the diagnoses of T2D has been described elsewhere (Nfor et al., 2018).

### Genotyping

The PNPLA3 rs738409 was genotyped using the custom Taiwan Biobank chip (Axiom™ Genome-Wide Array Plate System (Affymetrix, Santa Clara, CA, United States). The SNP call rate was >95% and was in Hardy-Weinberg equilibrium (*p*-value >1.0 × 10^–3^. The minor allele frequency (MAF) was >0.05.

### Statistical Analysis

Our data analyses were conducted with the software package PLINK 1.09 and the SAS software (version 9.4). We compared baseline parameters using the x^2^ test. We investigated the effects of T2D and *PNPLA3* rs738409 genetic variant on liver cirrhosis based on univariate and multivariate logistic regression analyses. The additive, dominant, and recessive models were used to determine the allele and genotype-specific ORs. Adjusted variables included sex, age, educational level, smoking, alcohol intake, body mass index (BMI), physical activity, hepatitis C virus (HCV), hepatitis B virus (HBV), and hyperlipidemia. In order to reduce potential bias, patients with cirrhosis before the diagnosis of diabetes were excluded from the final logistic regression models. We tested for interaction by including the term rs738409*T2D in the regression models. The ORs were estimated along with their 95% CI.

## Results

Table 1 describes the general characteristics of study participants. Overall, 150 of the 17,469 eligible participants were identified as cirrhosis cases. Among the cases of cirrhosis, 60 were of the rs738409 CC genotype, 62 were of the CG genotype, and 28 were of the GG genotype. About 21.33% (n = 32) of participants with liver cirrhosis had T2D. According to the univariate analysis, liver cirrhosis was positively associated with T2D (OR, 1.83; 95% CI 1.23–2.70) but the variant rs738409 was not despite the genetic model used (Table 2). Multivariate analyses, however, found no significant correlation between cirrhosis and rs738409 or T2D regardless of the model used. There was, however, a significant interaction between the variant and T2D in the additive, genotype, and dominant models (with *p* values of 0.0302, 0.0395, and 0.0455, respectively).

**TABLE 1:** Basic data of study participants.

	No liver cirrhosis	Liver cirrhosis	*p*-value
	N (%)	N (%)	
PNPLA3 rs738409			0.2504
CC	6,487 (37.13)	60 (40.00)	
CG	8,329 (47.68)	62 (41.33)	
GG	2,653 (15.19)	28 (18.67)	
Sex			0.0004
Female	9,049 (51.80)	56 (37.33)	
Male	8,420 (48.20)	94 (62.67)	
Age, year			<0.0001
30–39	4,932 (28.23)	17 (11.33)	
40–49	4,627 (26.49)	33 (22.00)	
50–59	4,752 (27.20)	58 (38.67)	
60–70	3,158 (18.08)	42 (28.00)	
Education			0.0454
Elementary School	1,002 (5.74)	8 (5.33)	
Junior/Senior High School	6,776 (38.79)	73 (48.67)	
University and above	9,691 (55.48)	69 (46.00)	
Cigarette smoking			0.0001
No	13,217 (75.66)	93 (62.00)	
Yes	4,252 (24.34)	57 (38.00)	
Alcohol drinking			0.0007
No	15,681 (89.76)	122 (81.33)
Yes	1788 (10.24)	28 (18.67)	
Body Mass Index (kg/m^2^)			0.6506
<18.5 and 18.5–24	8,927 (51.10)	71 (47.34)	
24–27	5,027 (28.78)	47 (31.33)	
≥27	3,515 (20.12)	32 (21.33)	
Exercise			0.0184
No	10,280 (58.85)	74 (49.33)	
Yes	7,189 (41.15)	76 (50.67)	
HCV			<0.0001
Negative	17,066 (97.69)	129 (86.00)	
Positive	403 (2.31)	21 (14.00)	
HBV			<0.0001
Negative	15,504 (88.75)	98 (65.33)	
Positive	1965 (11.25)	52 (34.67)	
T2D			0.0023
No	15,209 (87.06)	118 (78.67)	
Yes	2,260 (12.94)	32 (21.33)	
Hyperlipidemia			<0.0001
No	12,229 (70.00)	81 (54.00)	
Yes	5,240 (30.00)	69 (46.00)	

T2D, type 2 diabetes; HCV, hepatitis C virus; HBV, hepatitis B virus.

**TABLE 2:** Association of liver cirrhosis with PNPLA3 rs738409 and T2D.

PNPLA3 rs738409		Univariate	Additive model	Genotype model	Dominant model	Recessive model
		OR (95%CI)	aOR (95%CI)	aOR (95%CI)	aOR (95%CI)	aOR (95%CI)
Additive model	G vs. C	1.01 (0.80–1.28)	1.04 (0.82–1.31)	—	—	—
Genotype model	CG vs. CC	0.81 (0.56–1.15)	—	0.83 (0.58–1.18)	—	—
	GG vs. CC	1.14 (0.73–1.79)	—	1.19 (0.76–1.88)	—	—
Dominant model	GG + CG vs. CC	0.89 (0.64–1.23)	—	-	0.91 (0.66–1.27)	—
Recessive model	GG vs. CG + CC	1.28 (0.85–1.94)	—	—	—	1.32 (0.87–2.01)
T2D	Yes vs. No	1.83(1.23–2.70)	1.11 (0.72–1.71)	1.12 (0.72–1.73)	1.11 (0.72–1.71)	1.11 (0.72–1.72)
Sex	Male vs. Female	1.80 (1.29–2.52)	1.33 (0.89–1.99)	1.32 (0.88–1.98)	1.33 (0.89–2.00)	1.32 (0.88–1.98)
Age (years)						
40 to 49	40–49 vs. 30–39	2.07 (1.15–3.72)	1.7 (0.93–3.09)	1.69 (0.93–3.07)	1.70 (0.94–3.09)	1.69 (0.93–3.07)
50 to 59	50–59 vs. 30–39	3.54 (2.06–6.09)	2.76 (1.54–4.93)	2.76 (1.54–4.94)	2.76 (1.54–4.95)	2.75 (1.54–4.92)
60 to 70	60–70 vs. 30–39	3.86 (2.19–6.79)	3.07 (1.63–5.80)	3.05 (1.62–5.76)	3.08 (1.63–5.80)	3.06 (1.62–5.77)
Education						
Junior/Senior High School	ref: Elementary School	1.35 (0.65–2.81)	1.98 (0.93–4.21)	1.95 (0.91–4.15)	1.97 (0.93–4.20)	1.96 (0.92–4.18)
University and above	ref: Elementary School	0.89 (0.43–1.86)	1.63 (0.75–3.53)	1.61 (0.74–3.49)	1.63 (0.75–3.53)	1.62 (0.75–3.50)
Cigarette smoking	Yes vs. No	1.91 (1.37–2.65)	1.40 (0.93–2.10)	1.41 (0.94–2.12)	1.40 (0.93–2.10)	1.41 (0.94–2.12)
Alcohol drinking	Yes vs. No	2.01 (1.33–3.04)	1.31 (0.83–2.09)	1.31 (0.82–2.08)	1.31 (0.83–2.09)	1.31 (0.83–2.09)
Body Mass Index (kg/m^2^)						
<18.5	ref: 18.5 to 24	0.23 (0.03–1.65)	0.30 (0.04–2.20)	0.29 (0.04–2.14)	0.30 (0.04–2.20)	0.30 (0.04–2.16)
24 to 27	ref: 18.5 to 24	1.12 (0.77–1.63)	0.89 (0.60–1.30)	0.89 (0.60–1.30)	0.88 (0.60–1.30)	0.89 (0.61–1.30)
≥27	ref: 18.5 to 24	1.09 (0.72–1.66)	0.94 (0.60–1.45)	0.93 (0.60–1.45)	0.93 (0.60–1.45)	0.94 (0.60–1.45)
Exercise	Yes vs. No	1.47 (1.07–2.03)	1.13 (0.80–1.60)	1.13 (0.80–1.59)	1.13 (0.80–1.59)	1.13 (0.80–1.60)
HCV	Positive vs. Negative	6.89 (4.3–11.05)	5.87 (3.60–9.57)	5.79 (3.55–9.46)	5.83 (3.57–9.51)	5.86 (3.59–9.56)
HBV	Positive vs. Negative	4.19 (2.98–5.88)	4.55 (3.21–6.44)	4.56 (3.22–6.46)	4.54 (3.21–6.43)	4.56 (3.22–6.46)
Hyperlipidemia	Yes vs. No	1.99 (1.44–2.75)	1.39 (0.96–2.01)	1.39 (0.96–2.01)	1.39 (0.97–2.01)	1.39 (0.96–2.00)
*p*-value for interaction rs738409*T2D	—	0.0302	0.0395	0.0455	0.1872	

The ORs with their 95% CIs were estimated by a logistic regression model. The aORs with their 95% CIs were estimated by a multiple logistic regression model.

According to the univariate analyses, HCV and HBV infections both contributed significantly to cirrhosis risk, with ORs of 6.89 (95% CI, 4.3–11.05) and 4.19 (95% CI, 2.98–5.88), respectively. In spite of the analysis model, both variables remained significantly associated with liver cirrhosis as shown in Table 2. For subjects aged 40–49, 50–59, and 60–70, the ORs and 95% CIs were respectively 2.07 (1.15–3.72), 3.54 (2.06–6.09), and 3.86 (2.19–6.79) when compared with those aged 30–39. Our analysis of cases with and without T2D based on rs738409 genotypes (Table 3) indicated that diabetic individuals with the G allele had a higher cirrhosis risk compared to those with the C allele (OR, 1.71; 95% CI, 1.03–2.84), as did diabetic individuals with the GG genotype compared to those carrying the CC genotype (OR, 2.92; 95% CI, 1.07–7.99).

**TABLE 3:** Association of liver cirrhosis with rs738409 genotypes in individuals with and without T2D.

PNPLA3 rs738409		No T2D	T2D
		aOR (95%CI)	aOR (95%CI)
Additive model	G vs. C	0.90 (0.69–1.18)	1.71 (1.03–2.84)
Genotype model	CG vs. CC	0.71 (0.47–1.06)	1.63 (0.69–3.87)
	GG vs. CC	0.95 (0.56–1.60)	2.92 (1.07–7.99)
Dominant model	GG + CG vs. CC	0.77 (0.53–1.11)	1.92 (0.85–4.33)
Recessive model	GG vs. CG + CC	1.13 (0.69–1.84)	2.17 (0.95–4.95)

The aORs with their 95% CIs were estimated using multiple logistic regression models after controlling for sex, age, education, cigarette smoking, alcohol drinking, BMI, exercise, HBV, HCV, and hyperlipidemia.

## Discussion

Using the data of TWB participants collected from 2008 to 2015, we found that the *PNPLA3* gene variant rs738409 was not associated with liver cirrhosis while type 2 diabetes was. Despite these, we observed a significant interaction between the variant and T2D as shown in the additive, genotype, and dominant models. Furthermore, our analyses showed that rs738409 GG individuals who had T2D were more prone to liver cirrhosis than their CC counterparts.

Taiwan still faces a substantial number of liver diseases, including cirrhosis. It should be noted that cirrhosis is more prevalent among people with viral hepatitis (Mokdad et al., 2014). In contrast, 12.3–57% of patients with cirrhosis were also diagnosed with T2D (Trombetta et al., 2005). In a recent review, Lee and colleagues claimed that there is a higher prevalence of diabetes in patients with liver cirrhosis than previously believed (Lee et al., 2019). Numerous studies have already suggested a link between liver cirrhosis and T2D. However, there is little data about genetic links between these two health conditions. To gain more insight into the relationship, we replicated the rs738409 variant and showed that it may interact with T2D to increase cirrhosis risk. This variant showed associations with liver disease, T2D, and other disorders in diverse populations as mentioned above. Additionally, it has been linked to non-viral hepatitis-associated hepatocellular carcinoma among T2D patients (Ueyama et al., 2016).

The relationship between viral hepatitis and liver cirrhosis is well known. Our univariate and multivariate models also showed that participants with HBV and HCV infection had an increased risk for cirrhosis. Cirrhosis is believed to develop in most viral hepatitis patients around the age of 65 or so (Pradat et al., 2007). Our study found that cirrhosis prevalence was higher among individuals aged 50 to 59 and 60 to 70.

Our study has the advantage of a large sample size. By using personal identification numbers, we were able to link genetic, clinical, and lifestyle information from the TWB database to medical records from the NHIRD; these data enabled more detailed and accurate analyses at the individual level. Our study was limited, however, by the lack of information regarding the severity of liver cirrhosis. There is a need for further research into the role of T2D and *PNPLA3* rs738409 in the development of liver cirrhosis.

This study indicates that Taiwanese adults with T2D who have the *PNPLA3* rs738409GG genotype might have a higher risk for liver cirrhosis than those with the CC genotype. Findings like these contribute to a better understanding of the genetic bases of liver cirrhosis, which may be useful in personalized medicine.

## Data Availability

The original contributions presented in the study are included in the article/Supplementary Material, further inquiries can be directed to the corresponding authors.
